# Effects of Processing Parameters of Selective Laser Melting Process on Thermal Conductivity of AlSi10Mg Alloy

**DOI:** 10.3390/ma14092410

**Published:** 2021-05-06

**Authors:** Moo-Sun Kim

**Affiliations:** Urban Transit Research Team, Korea Railroad Research Institute, Uiwang 16105, Korea; mskim@krri.re.kr

**Keywords:** thermal conductivity, anisotropic, SLM, scan speed, hatch spacing, cell structure

## Abstract

Selected laser melting (SLM) is a representative process of powder-bed type 3D printing technology that is used to manipulate metals and it generally results in various structural properties according to the process conditions. In this study, a thermal conductivity test was conducted on AlSi10Mg specimens that were manufactured using the SLM process to investigate the influence of various process conditions on the thermophysical characteristics and cellular microstructure of the samples. The building direction of the specimen, laser scan speed, and hatch spacing were considered as process variables, and the thermal conductivity was analyzed for each process variable. In the test results, as the polar angle of the specimen increased from 0° to 90°, the thermal conductivity increased. Furthermore, the thermal conductivity gradually decreased as the scan speed and hatch spacing increased. The differences in thermal characteristics are discussed in connection with the microstructure of the cells constituting the melt pool. The solidified melt pool that formed by the laser beam is composed of cells consisting of an Al matrix and a surrounding Si-rich area. The differences in thermal conductivity of the overall specimens are explained based on the variations in thermal conductivity and cell shape and size for each chemical component.

## 1. Introduction

Additive manufacturing (AM) technology is a state-of-the-art manufacturing process technology that creates 3D structures by repeatedly stacking 2D planar structures. AM can dramatically reduce the number of manufacturing steps by enabling the integral manufacturing of complex-shaped parts that require several steps when manufactured using traditional methods, such as welding and assembly [1]. It is a recent manufacturing technology that enhances performance and efficiency by increasing the product design freedom. In addition, topology optimization technology, which has been used as a lightweight implementation method to design concept products, can be used as a design method to realize products [2]. Owing to these advantages, the field of AM technology application as a product manufacturing technology is gradually expanding; in fact, in industries, such as aviation, automobile, and medicine, it is applied to the production of actual commercial products.

Currently, various techniques, such as selective laser melting (SLM), direct metal deposition (DMD), and the electron beam melting (EBM) process, are being developed for AM [3]. Among them, the SLM process is a representative technology that is used for the mass manufacture of metal products. In SLM, a three-dimensional structure is fabricated by repeatedly melting and solidifying selected two-dimensional areas and a high-power laser beam is used to melt metal powder with a diameter of several tens of micrometers. The material is dispersed on a two-dimensional plane and fused to the lower layered structure. Currently, metal materials that can be used in the SLM process include titanium, aluminum alloy, stainless steels alloys, and nickel alloys, and research and development for further materials are actively underway.

Because the SLM process uses a laser beam to fuse materials, it has the advantage of achieving excellent physical properties. However, the quality of the final product is sensitive to laser irradiation conditions and material powder properties. Therefore, research on the analysis of changes in physical properties according to process conditions, such as laser irradiation and powder properties, must be performed.

Olakanmi [4] showed that the laser power and scan speed are the most influential processing parameters in the process window for avoiding the agglomeration of powders and obtaining a continuous deposition surface in the SLM process while using aluminum alloy powder. Aboulkhair et al. [5] studied the effect of the hatch spacing and scan speed on the porosity of AlSi10Mg and optimized those conditions to achieve the highest density by reducing the pores in the SLM process.

Shifeng at al. [6] explained the anisotropy of the mechanical properties of structures that are manufactured via the SLM process by considering the influence of melt pool boundaries on microscopic slipping during loading. Anwar and Pham [7] studied the change in tensile strength as a function of the scan direction of the SLM process, the position of the fabricated specimen for aluminum alloys, and the inert gas flow velocity. Hitzler et al. [8] investigated the change in tensile properties based on the combination of the building orientation and position of the specimen on the fabrication bed. They showed that the results for the tensile and hardness tests have similar tendencies for samples without heat treatment.

Other studies focused on the thermophysical properties of metal structures that are produced via the SLM process. Alkahari et al. [9] conducted a comparative analysis of the thermophysical properties of SUS and Cu-Ni alloys in powder form and the thermophysical properties of specimens that are produced via the SLM process. The results indicated that the difference in heat conductivity occurred, owing to the influence of the density, particle size, and porosity of metal materials. Yang et al. [10] explained the low thermal diffusivity and thermal conductivity of an as-built AlSi10Mg part through the formation of supersaturated primary α aluminum and unique cellular structure and established a correlation between microstructural evolution and thermal properties. Strumza et al. [11] analyzed the effect of texture characteristics and internal pore distribution on the anisotropic thermophysical properties on the x-axis and z-axis for AlSi10Mg and explained the enhancement of thermophysical properties through high-temperature heat treatment.

In terms of research on the effects of laser irradiation on the microstructure of the melt pool, Maity et al. [12] showed that variation in the local mechanical properties is due to the local variations in the microstructure according to the position in the melt pool. Liu et al. [13] investigated the influence of laser power in SLM on the grain morphology and texture components in AlSi10Mg alloy, and explained the formation of three different zones that were observed in the melt pool caused by the difference in the morphology of the Si phase. Prashanth et al. [14] considered the effects of cell microstructure, according to the heat treatment temperature on the mechanical properties of Al-12Si, and showed that mechanical strength and ductility can be tuned by varying the microstructure according to the proper annealing treatment. Liu et al. [15] demonstrated the influence of the remelting strategy on the microstructure in the melt pool of AlSi10Mg alloy and showed that the remelting strategy improves the surface quality and relative density of the AlSi10Mg sample. Hyer et al. [16] examined the microstructure characteristics of AlSi10Mg in terms of various processing parameters, such as laser power, hatch spacing, and scan speed. They found the proper energy density range to produce the highest density samples and concluded that the sub-grain cellular structure decreases in size with increasing scan speed and increasing slice thickness.

AlSi10Mg is a representative material for parts, such as heat exchangers in transportation vehicles, and it constitutes a good application of the SLM process and topology optimization, owing to its light weight and excellent thermal performance. Therefore, it is necessary to understand the correlation between the process conditions and the heat transfer characteristics of AlSi10Mg alloy in order to maximize the performance of SLM-manufactured parts in terms of their heat transfer characteristics.

In this study, thermal conductivity, which is a key parameter in designing heat transfer parts, was measured for an AlSi10Mg specimen that was manufactured using the SLM process. The building direction, laser scan speed, and hatch spacing were considered to be the main parameters among the SLM process conditions. The differences in thermal conductivity were also studied through analysis of the change in morphology and size of the cellular microstructure in the melt pool generated by the laser beam according to the test processing parameters.

## 2. Methodology

### 2.1. Fabrication of Thermal Conductivity Test Specimen

The thermal conductivities of AlSi10Mg alloy specimens were analyzed for different SLM process conditions. The specimens were manufactured using SLM with the EOS M290 machine (EOS GmbH, Munich, Germany). The M290 uses a 400 W Yb-fiber laser with a laser spot diameter of 100 μm, and the laser scan speed can reach up to 7.0 m/s. The material for the specimen production was AlSi10Mg, an aluminum alloy material that was obtained from EOS. Table 1 lists the chemical composition of the specimens, as given in the material provider’s data sheet [17]. The sizes of the specimens were 10 mm × 10 mm × 3 mm. The process parameters that were considered included the laser scan speed and hatch spacing, as well as the direction in which each specimen was built.

**Figure 1:**
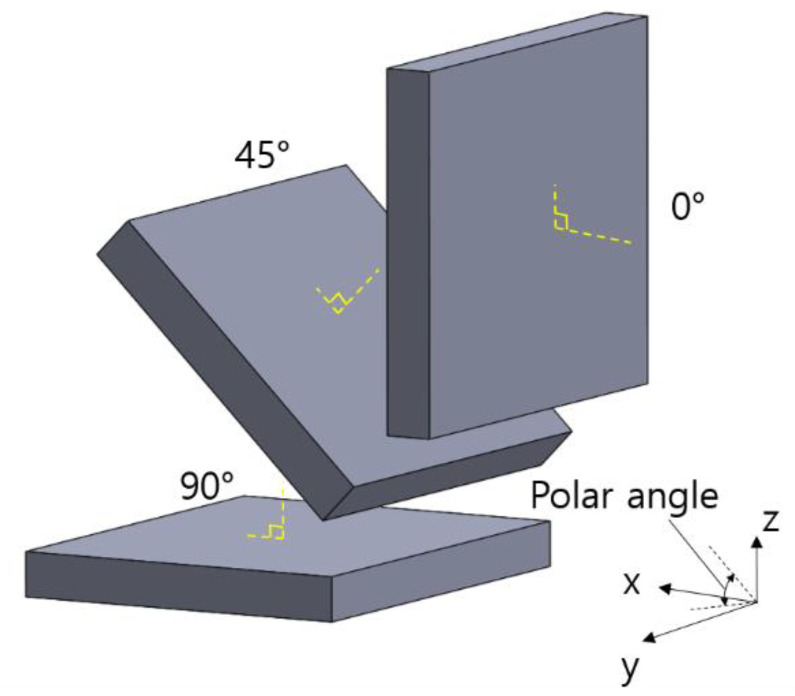
Schematic description of thermal conductivity test samples on the substrate plate.

The thermal conductivity specimen is produced on the substrate plate in the SLM machine when classifying by the specimen building direction. When the substrate plate plane is defined as the xy-plane, and the angle between the xy-plane and the axis perpendicular to the wide plane of the specimen is defined as the polar angle. The specimens were prepared with polar angles of 0°, 45°, and 90°. Figure 1 shows the specimen shape at different polar angles. In the thermal conductivity test, the specimen with a polar angle of 0° exhibits the thermal characteristics in the xy-plane, whereas the 90° specimen shows the thermal characteristics in the z-axis.

**Figure 2:**
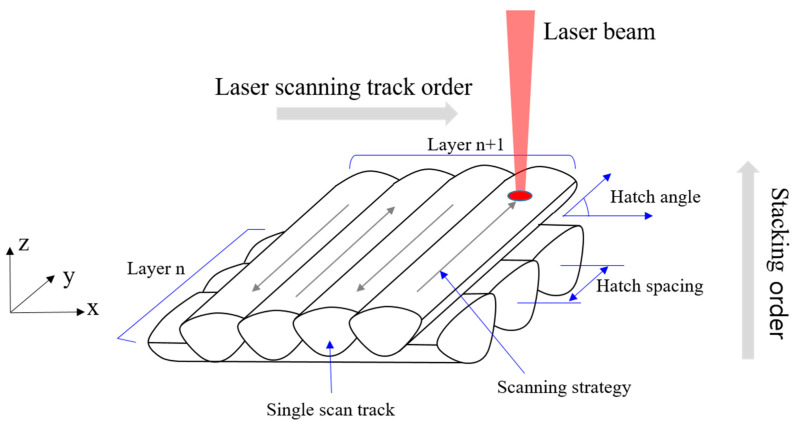
Concept of laser irradiation in the SLM process.

The laser scan speed, which is, the speed at which the laser passes through while melting the powder, was considered as another process variable. Figure 2 shows the concept of laser irradiation conditions to be considered when forming each layer of a structure in the SLM process. The hatch spacing represents the interval between adjacent same-layered single tracks that were scanned by the laser, and the hatch angle represents the angle between the scanning vectors of layers n and n + 1. The scanning strategy defines the laser scanning pattern.

Among the methods of characterizing the process conditions in SLM, the simplest and most important approach is to consider the energy density [4]. The energy density is closely related to the properties of the melt pool that are generated to fuse the material powder to the lower layer structure through laser irradiation. The mathematical definition of the energy density is expressed as Equation (1).

(1) $$E=\frac{P}{v\cdot h\cdot t}$$

where *E*, *P*, *v*, *h*, and *t* represent the energy density function (J/mm^3^), laser power (W), laser scan speed (mm/s), hatch spacing (mm), and layer thickness (mm), respectively.

The laser power was kept constant at 285 W to manufacture the thermal conductivity test specimen. The scan speed and hatch spacing for the standard test case were fixed to 1700 mm/s and 0.12 mm, respectively, referring to the optimum process conditions proposed by the equipment manufacturer. The value of the scan speed as a process variable was determined in intervals of 10% starting from 1700 mm/s. Thus, the test was divided into three scan speed cases: 1530 mm/s, 1700 mm/s, and 1870 mm/s. The hatch spacing condition was also divided into three cases: 0.1 mm, 0.12 mm, and 0.14 mm. Moreover, the added layer thickness was kept constant at 30 μm, and the hatch angle was set to 67°. The substrate plate was prepared in a pre-heating state to maintain its temperature at 150 °C.

Table 2 lists the process condition values for each test case for different manufacturing process conditions; further, the calculated energy density corresponding to each process condition is shown.

### 2.2. Test Procedure

The density, heat capacity, and thermal diffusivity were measured to obtain the thermal conductivity of the SLMed specimen. Subsequently, the thermal conductivity was calculated using Equation (2) [18].

(2) $$K=\rho C_{p}\alpha ,$$

where *K* (W/(m·K)), *ρ* (kg/m^3^), *C_p_* (J/kg/K), and *α* (mm^2^/s) denote the thermal conductivity, density, heat capacity, and thermal diffusivity, respectively.

The test specimen manufactured using SLM was adjusted to a thickness of 2 mm through surface grinding. The bulk density at a temperature of 25 °C was measured using Archimedes’ principle and it was corrected by considering anisotropic thermal expansion. Thermal mechanical analysis (Q400 from TA Instrument, New Castle, DE, USA) was used to determine thermal expansion along the test polar angle in a temperature range from 0 °C to 250 °C. The heating rate was 5 °C/min. under flowing nitrogen, and the applied force was 0.02 N. The TMA was calibrated with an aluminum cylinder standard and error was below 1%.

Differential scanning calorimetric analysis was performed to measure the heat capacity. DSC8000 from Perkin Elmer (Waltham, MA, USA) was used in a temperature range from 5 °C to 220 °C at a heating rate of 20 °C/min. under a 25-mL/min. flow of nitrogen. The heat capacity was calibrated to a sapphire standard and it showed an average error below 0.6%.

A thermal diffusivity test was performed using a laser flash apparatus (LFA447 Nanoflash instrument from NETZSCH-Geratebau GmbH, Selb, Germany), and the data were collected after exposing the specimen for 5 min. at the designated temperature.

### 2.3. Microstructure Characterization

An optical microscope (OM, BX51M, Olympus, Tokyo, Japan) and a scanning electron microscope (SEM, JSM-6510, JEOL Ltd, Tokyo, Japan) were used to observe the melt pool shape and microstructure characteristics of the samples. The samples were finally polished with diamond powder after performing the conventional grinding and polishing steps and they were etched by Keller’s solution (190 mL H_2_O, 5.0 mL HNO_3_, 3.0 mL HCl and 2.0 mL HF) [13]. TEM/EDS analysis was performed by cutting the specimen from the melt pool core and boundary for the polar angle 0° specimen. The analysis equipment comprised the JEOL JEM-ARM200F (Tokyo, Japan).

## 3. Results and Discussion

Table 3 summarizes the average density results of the specimens for each test case and polar angle. The density measurements show differences of 0.2%, at most, according to the process condition and polar angle.

**Figure 3:**
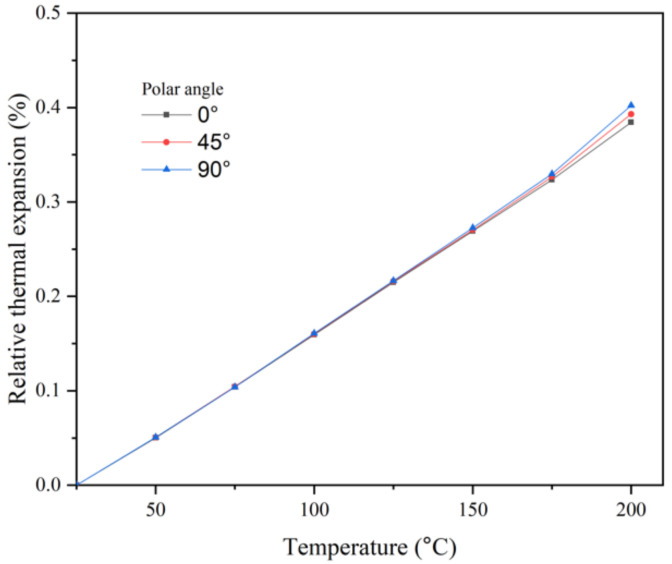
Relative thermal expansion for case 2 as a function of the polar angle.

In Figure 3, the results of relative thermal expansion are presented as a function of the polar angle for case 2. The difference in thermal expansion for all polar angles gradually increases, as the temperature increases. The thermal expansion results show a linear correlation with the temperature and almost no dependence on the polar angle up to approximately 120 °C. For higher temperatures, the thermal expansion of the polar angle 90° shows slight upward deviation when compared to the results of polar angle 0° and 45° specimens.

**Figure 4:**
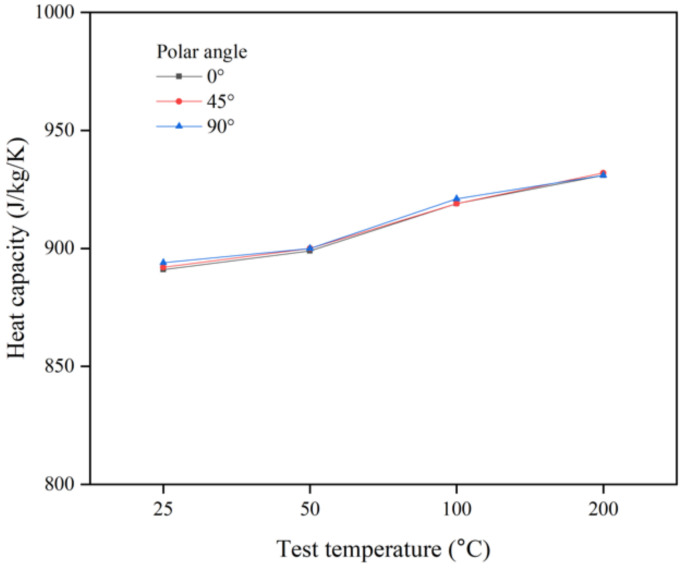
Heat capacity for case 2 as a function of the polar angle at test temperatures of 25, 50, 100, and 200 °C.

Figure 4 shows the *C_p_* results obtained from DSC analysis as a function of the polar angle for case 2, which is a reference process case, at the designated test. The values of *C_p_* for each polar angle increase slightly, and no significant difference is observed as a function of the polar angle, as it is a volumetric value [11].

**Figure 5:**
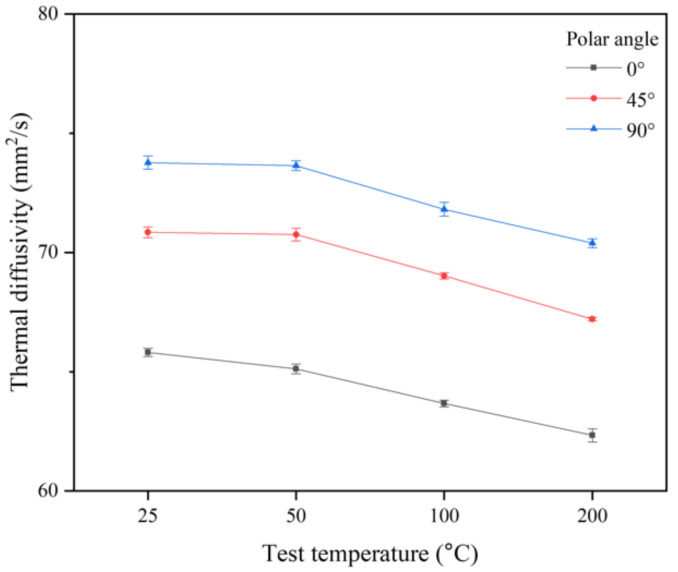
Thermal diffusivity for case 2 as a function of the polar angle at test temperatures of 25, 50, 100, and 200 °C.

Figure 5 shows the thermal diffusivity results as a function of the polar angle for case 2 at the designated test temperature. The dependency on the polar angle can be clearly confirmed and is in accordance with the qualitative results presented in other studies [10,11]. Based on the test temperature of 25 °C, the average value of the thermal diffusivity of the polar angle 90° specimen is approximately 12.3%, which is 4.2% higher than the corresponding values for the polar angle 0° and 45° specimens, respectively.

**Figure 6:**
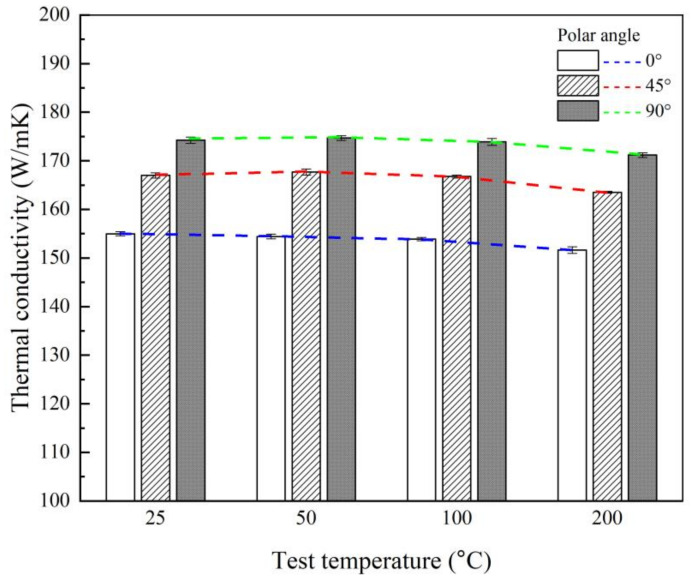
Comparison of thermal conductivity for case 2 as a function of the polar angle at test temperatures of 25, 50, 100, and 200 °C.

Figure 6 shows the thermal conductivity results calculated using Equation (2) for case 2. Under all test temperature conditions, the larger the polar angle is, the larger the thermal conductivity value is, and the tendency is similar to that of the thermal diffusivity results. Based on the test temperature of 25 °C, the average thermal conductivity value of the polar angle 90° specimen is 174.2 W/(m·K), which is 12.4% and 4.3% higher than the corresponding values for the polar angle 0° and 45° specimens, respectively. For test temperatures of 50 °C, 100 °C, and 200 °C, the thermal conductivity gradually decreases with increasing temperature, and the differences in thermal conductivity as a function of the polar angle show a similar pattern. Regarding the anisotropy of thermal characteristics, the thermal conductivity along the z-axis is relatively higher than that in the xy-plane in the test structure that is manufactured via the SLM process.

**Figure 7:**
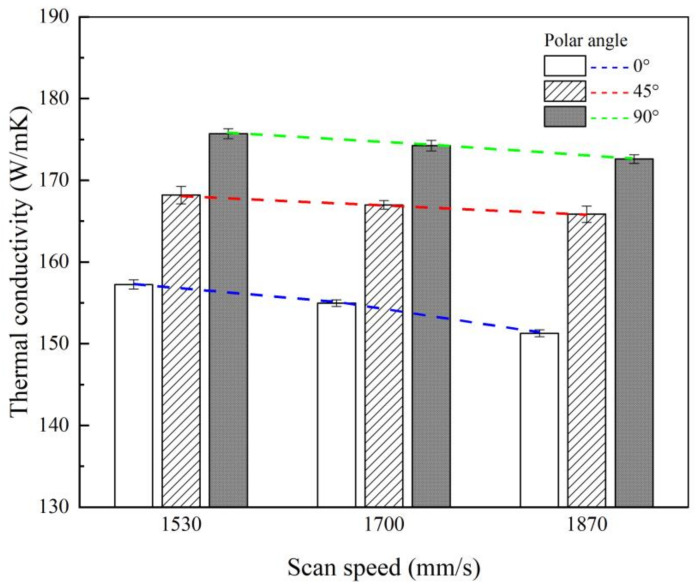
Comparison of thermal conductivity as a function of the laser scan speed at a test temperature of 25 ℃.

As one of the main processing conditions, the effects of laser scan speed on the thermal conductivity were considered. Figure 7 shows the thermal conductivity results at a test temperature of 25 °C for each polar angle and scan speed with hatch spacing of 0.12 mm and other conditions kept constant. First, under all scan speeds, it is shown that, the larger the polar angle, the larger the thermal conductivity. The thermal conductivity decreases as the scan speed increases in all polar angle cases. At a scan speed of 1870 mm/s, the average thermal conductivity decreased by approximately 3.3% from 156.5 W/(m·K) to 151.2 W/(m·K) in the case where the polar angle is 0° as compared to the thermal conductivity under the scan speed condition of 1530 mm/s. When the polar angle was 90°, the average thermal conductivity decreased by approximately 1.4% from 174.9 W/(m·K) to 172.5 W/(m·K). The lower the polar angle, the larger the change in the thermal conductivity. In terms of energy density, the smaller the energy density, which is inversely proportional to the scan speed, the smaller the thermal conductivity.

**Figure 8:**
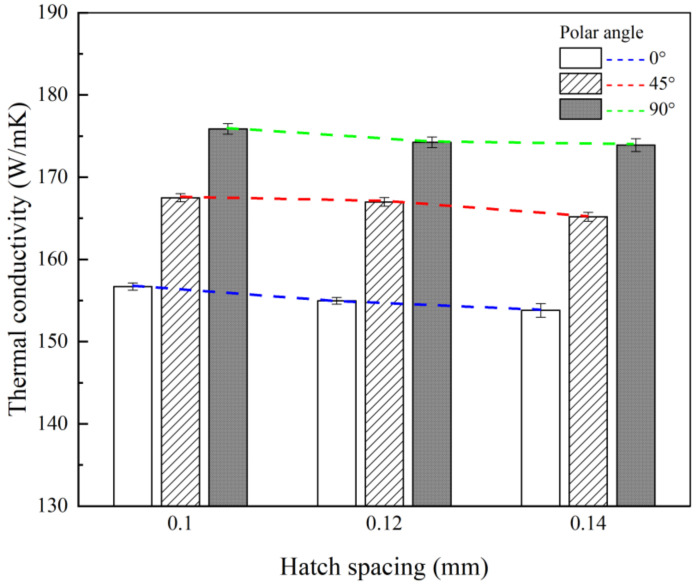
A comparison of thermal conductivity as a function of the hatch spacing at a test temperature of 25 ℃.

As another processing variable, the thermal conductivity values were compared for different hatch spacing values. Figure 8 shows the thermal conductivity results for the specimens that were manufactured with various hatch spacing values and polar angles at a scan speed of 1700 mm/s and test temperature of 25 °C; other processing conditions were kept fixed. As with previous results, as the polar angle increases in each hatch spacing condition, the thermal conductivity increases. Further, it is shown that the thermal conductivity gradually decreases as the hatch spacing increases. At a polar angle of 0°, the average thermal conductivity for a 0.14-mm hatch spacing decreases by approximately 2.2% from 156.7 W/(m·K) to 153.2 W/(m·K) as compared to that for a 0.1-mm hatch spacing. At a polar angle of 90°, the average thermal conductivity for a 0.14-mm hatch spacing decreases by approximately 1.5% from 175.8 W/(m·K) to 173.2 W/(m·K) when compared to that for a 0.1-mm hatch spacing. It seems that, as the polar angle increases, the change in the thermal conductivity that is caused by increasing the hatch spacing decreases slightly. Furthermore, as in the case of the scan speed, the smaller the energy density, which is inversely proportional to the hatch spacing, the smaller the thermal conductivity.

From the test results, it was confirmed that the difference in thermal conductivity followed the building direction of the specimen and the main processing conditions of laser scan speed and hatch spacing. Several studies [6,7,8,12,13,14] explained the differences in mechanical properties in terms of the differences in the microstructure that were observed inside the melt pool, the progression direction of the melting track, and extremely fast undercooling in the SLM process. We analyzed the reason for the anisotropy of thermal properties and the differences in thermal properties according to process conditions when considering the microstructure inside the melt pool.

The melt pool is created when the material on the powder bed in the SLM machine is irradiated by a laser. Melt pools are continuously created following the laser scanning direction, as shown in Figure 2. Figure 6 shows the shape of the melt pool group on the wide plane of a test specimen with at each polar angle.

**Figure 9:**
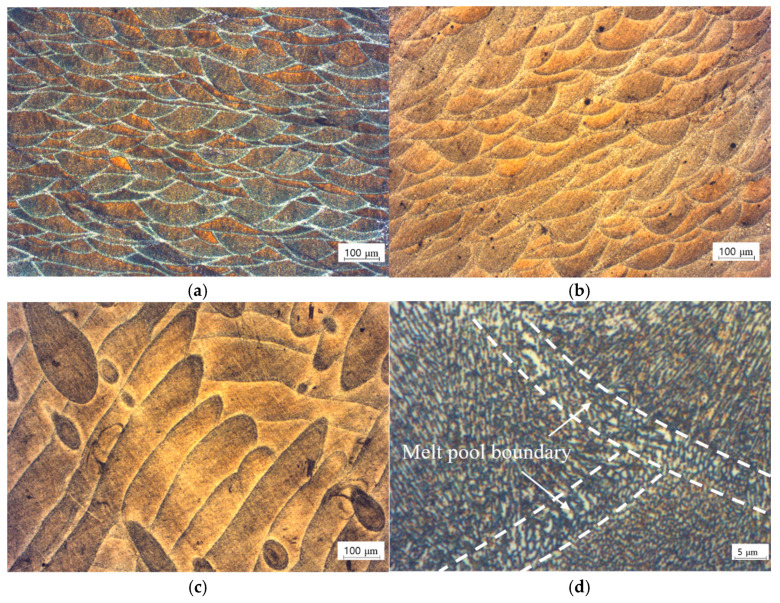
Optical microscope image of melt pool on the wide surface of a specimen from case 2 with polar angles of (**a**) 0°, (**b**) 45°, and (**c**) 90°; and, (**d**) microstructure inside melt pool.

In Figure 9, in the cross-section of the specimen with a polar angle of 0°, it is observed that a hemispherical melt pool with a similar shape of a Gaussian distribution of the laser beam power is repeatedly generated as it overlaps the neighboring melt pool. This pool has an elongated elliptical shape in the longitudinal section of the melt pool track along the laser scan direction in the specimen with a polar angle of 90°.

A melt pool is composed of the white cellular structures of the Al matrix and the eutectic fibrous Si network surrounding the Al matrix, as shown in Figure 9d. These cell compositions are the same as those found in many studies [12,13,14,15,16,19,20,21,22] on the inner cell structure of the melt pool in the SLM process. Constitutional undercooling is required to create a cellular structure [19]. As the liquid phase melt pool solidifies, the Si concentration in the liquid increases, owing to the discharge of Si from the solidifying front. Therefore, the Al matrix solidifies first in the cell form as a result of the high cooling rate and solubility of Si, and Si is distributed around it to form a cell boundary.

In the overlapping section of the laser scan tracks, the boundary of the melt pool is generated, and the cellular structure that is visible in this area is coarser in size than the cellular structure that s found in the melt pool core, owing to the influence of overheat by remelting. This difference in cell size originates from the thermal gradient *G* and the solidification rate *R* [15,16]. The fineness of the cellular structure can be estimated from the product of *G* and *R*; the higher the value of this product, which is related to the cooling rate, the finer the cellular structure. In contrast, the value of *G* divided by *R* is related to the solidification mode. *Cst,* which is defined as the function of nucleant density and equilibrium liquids-solidus interval temperature [23], is the criterion of columnar to equiaxed transition in the cellular structure shape [23,24]. When the ratio of *G* to *R* exceeds this value, a columnar dendritic structure is predicted and, when the ratio is smaller than *Cst*, an equiaxed dendritic structure is predicted. In the SLM process, *G* and *R* are diversified, even inside melt pools that are generated by the laser scan owing to the movement of the laser beam, which is a heat source. That is, the *G* and *R* values are higher at positions closer to the melt pool center and lower toward the melt pool boundary. Therefore, the coarse cell at the edge of the melt pool becomes finer as it approaches the melt pool center. In addition, as the cell near the melt pool boundary undergoes solidification first at the solid-liquid interface, equiaxed cells are formed, whereas the cells grow in the same direction as the thermal gradient distributed radially from the center of a melt pool in the melt pool core. Thus, columnar cellular structures are shown in the melt pool core [20].

**Figure 10:**
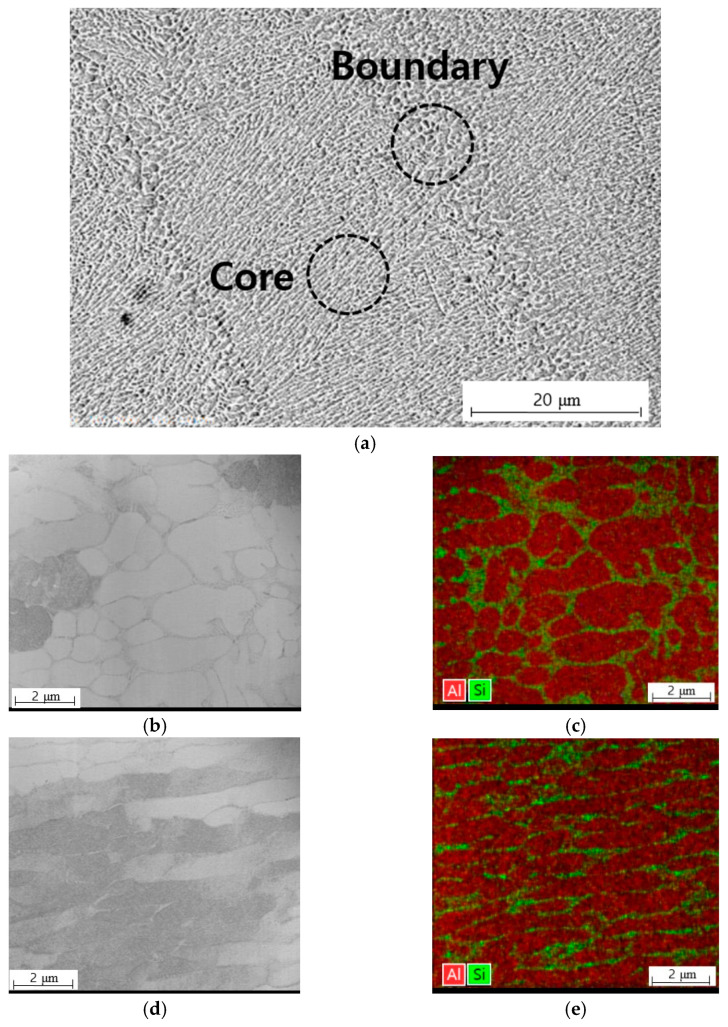
(**a**) SEM representative image of melt pool; (**b**) morphology of microstructure by TEM and (**c**) EDX composition map at boundary of melt pool; (**d**) morphology of microstructure by TEM; and, (**e**) EDX composition map at core of melt pool.

Figure 10 shows the cellular structure shape and chemical composition map for each melt pool core and boundary. The cellular structure that is distributed in the melt pool boundary is close to the form of an equiaxed one shown in Figure 10b, and most of the cellular structures that are distributed in the melt pool core have a column shape in Figure 10d. In both cases, it can be observed that the structure is surrounded by an Al matrix and fibrous Si network in Figure 10c,e.

It has been confirmed that the mechanical properties of AlSi10Mg that are produced via SLM depend on the elongated orientation and size of the cellular structure observed above [19,21,22]. The difference in mechanical properties is related to the cell characteristics of the Al matrix and surrounding Si-rich region in the cellular structure. Further, it can be reduced by converting the Si-rich region in the cell boundary into Si particles by performing heat annealing at the proper temperature [19]. Likewise, the correlation between the morphology and size of cell structures and thermal properties is required to explain the difference in thermal properties depending on the process conditions.

The general thermal conductivity of Al is approximately 237 W/(m·K), and that of Si is approximately 148 W/(m·K) [25]. The thermal properties of AlSi10Mg alloy can generally be predicted from the homogenization of the thermal properties of each component material; however, in SLM-manufactured products, it is necessary to consider the characteristics of the various microstructure separated by the Al matrix and Si region in the melt pool. That is, thermal conductivity may vary, depending on the distribution patterns of Al and Si, which have different thermal properties.

When considering the shape of the cellular microstructure in terms of thermal characteristics, the Si-rich region with relatively low thermal conductivity surrounds the Al matrix with high thermal conductivity. This means that heat is rapidly transferred through the Al matrix, whereas the heat transfer is slower in the Si-rich region, owing to low thermal conductivity.

**Figure 11:**
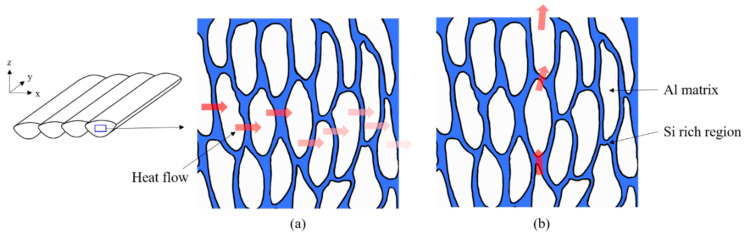
Example of heat flow through cells in melt pool core along (**a**) x-axis and (**b**) z-axis.

In addition, as cell growth proceeds toward the core in the melt pool, the heat transfer in the longitudinal direction of the cell increases as the length of the Al matrix with high thermal conductivity increases. Figure 11 shows an example of an area inside the melt pool. When considering a Si-rich region with relatively low heat conductivity in the heat transfer path, the number of Si-rich regions along the heat flux direction increases along the xy-plane, rather than the z-axis, because cells grow in the z-axis direction. This means that more heat flux proceeds along the z-axis than along the xy-plane.

At the boundary of the melt pool, there exists a coarse cellular zone consisting of cells, which have equiaxed structures and are coarser than cells inside the melt pool core. The heat affected zone (HAZ) also exists at the outside of the coarse cellular zone; the HAZ has a shape, in which the fibrous Si network is broken into particles [19]. From these structural characteristics, it can be expected that the high thermal conductivity of the Al matrix is more effective at the melt pool interface than in the melt pool core region.

When linking the cell structural characteristics with the thermal conductivity test results for different polar angles, it is found that the thermal conductivity of the specimen with a polar angle of 90° is higher than that with a polar angle of 0°. This is because the former specimen contains less Si-rich boundaries of cells in the melt pool core region and it has more melt pool boundaries in the thermal conductivity test area along the test direction.

When considering the effect of the laser scan speed on the change in the cellular microstructure, Hyer et al. [16] experimentally confirmed that. the higher the scan speed, the smaller the cell size. Thus, as the scan speed decreases, the energy density increases and the cooling rate drops. Subsequently, the cell size increases [20], and the number of Si rich boundary regions with low thermal conductivity in the melt pool decreases, leading to higher heat flow than that in the finer cell region. In addition, as the regions of the coarse cellular zone and the HAZ of the melt pool interface become relatively larger. owing to the high energy density, the overall heat conductivity is expected to increase. This agrees with the results of the thermal conductivity test as a function of the scan speed.

**Figure 12:**
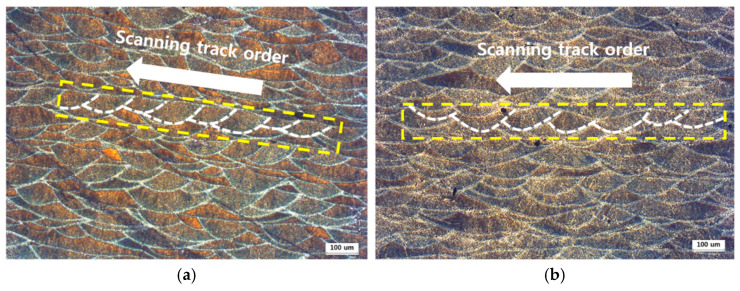
Shape of overlapped tracks with hatch spacing of (**a**) 0.1 mm and (**b**) 0.14 mm.

Hatch spacing is related to the generation period of the melt pool and the size of the area overlapping with the neighboring track. Figure 12 shows the overlapped track pattern of the melt pool for two cases with hatch spacings of 0.1 mm and 0.14 mm. It is shown that, the shorter the hatch spacing, the shorter the repeating period of the same-size melt pool. This means that the number of melt pool boundary regions increases in a specified size area, and relatively high heat conductivity can be expected as the number of boundary regions that are composed of coarse cells increases, because the heat conduction characteristics of the Al matrix are more heavily reflected in these areas. Further, the smaller the hatch spacing, the higher the energy density and the larger the cell size, thereby enhancing the heat transfer effect. This is consistent with the test results showing that the shorter the hatch spacing is, the higher the thermal conductivity is.

## 4. Conclusions

In this study, the thermal conductivity of AlSi10Mg alloy specimens manufactured using SLM technology was analyzed under various processing conditions. The change in the thermal conductivity was observed by varying the specimen polar angle, laser scan speed, and hatch spacing. Further, the cause of the difference in the thermal conductivity was explained based on the characteristics of the melt pool and the internal cellular microstructure. The results of this analysis are summarized, as follows.

- As the polar angle, which is dependent on the building direction of the specimen, increases (that is, the direction becomes closer to the z-axis), the heat conductivity in the polar angle direction increases. This is believed to be related to the orientation of the melt pool and the cellular structure inside the melt pool. Cells that consist of an Al matrix that is surrounded by a Si-rich region grow radially from the center of the melt pool core area, and the fibrous Si network is broken at the boundary of the melt pool to widen the Al matrix area. As a cell is formed, the heat conductivity increases in the z-axis direction, to which the growth direction of the Al matrix (which has relatively higher heat conductivity) is close, and more melt pool boundary regions in the path of the heat flow along the z-axis are included.
- The thermal conductivity decreases as the scan speed increases and, in turn, the energy density increases. Further, as the cooling rate decreases, the size of the cell structure inside the melt pool increases. Consequently, the number of cells with a Si-rich boundary with low thermal conductivity in the melt pool decreases and, thus, there is more heat flow than in the melt pool with the finer cells.
- The shorter the hatch spacing, the higher the thermal conductivity. This is related to the repetition period of the melt pool generation and the increase of the cell size as the energy density increases. As the period of melt pool generation is shortened, the number of the melt pool boundary with high thermal conductivity increases, and the cell size also increases, owing to the increase in energy density. Further, as the influence of the Si-rich region around the cell decreases, the thermal conductivity increases.

## Figures and Tables

**Table 1:** Chemical composition of AlSi10Mg powder (wt.%).

Si	Fe	Cu	Mn	Mg	Ni	Zn	Pb	Sn	Ti	Al
9.0–11.0	≤0.55	≤0.05	≤0.45	0.2–0.45	≤0.05	≤0.10	≤0.05	≤0.05	≤0.15	Balance

**Table 2:** SLM process conditions to manufacture thermal conductivity specimens for each test case.

Case Number	Laser Power (W)	Scan Speed (mm/s)	Hatch Spacing (mm)	Energy Density (J/mm^3^)	Polar Angle (°)
1	285	1700	0.1	55.88	0, 45, 90
2		1700	0.12	46.57	
3		1700	0.14	39.92	
4		1530	0.12	51.74	
5		1870	0.12	42.34	

**Table 3:** Average density of specimens in each case according to polar angle.

Case Number	Average Density (kg/m^3^, with Standard Deviation ≤0.1%)		
	0°	45°	90°
1	2646 2643 2641 2645 2643	2646 2642 2641 2643 2642	2643 2642 2640 2645 2640
2			
3			
4			
5			

## Data Availability

The data presented in this study are available on request from the corresponding author.

## References

[1] Olakanmi E.O., Cochrane R.F., Dalgarno K.W. (2015). A review on selective laser sintering/melting (SLS/SLM) of aluminum alloy powders: Processing, microstructure, and properties. Prog. Mater. Sci..

[2] Zhu J., Zhou H., Wang C., Zhou L., Yuan S., Zhang W. (2021). A review of topology optimization for additive manufacturing: Status and challenges. Chin. J. Aeronaut..

[3] Gadagi B., Lekurwale R. (2020). A review on advances in 3D metal printing. Mater. Today Proc..

[4] Olakanmi E.O. (2013). Selective laser sintering/melting (SLS/SLM) of pure Al, Al–Mg, and Al–Si powders: Effect of processing conditions and powder properties. J. Mater. Process. Technol..

[5] Aboulkhair N.T., Everitt N.M., Ashcroft I., Tuck C. (2014). Reducing porosity in AlSi10Mg parts processed by selective laser melting. Addit. Manuf..

[6] Shifeng W., Shuai L., Qingsong W., Yan C., Sheng Z., Yusheng S. (2014). Effect of molten pool boundaries on the mechanical properties of selective laser melting parts. J. Mater. Process. Technol..

[7] Anwar A.B., Pham Q. (2017). Selective laser melting of AlSi10Mg: Effects of scan direction, part placement and inert gas flow velocity on tensile strength. J. Mater. Process. Technol..

[8] Hitzler L., Janusch C., Schanz J., Merkel M., Heine B., Mack F., Hall W., Ochsner A. (2017). Direction and location dependency of selective laser melted AlSi10Mg specimens. J. Mater. Process. Technol..

[9] Alkahari M.R., Furomoto T., Ueda T., Hosokawa A., Tanaka R., Aziz M.S.A. (2012). Thermal conductivity of metal powder and consolidated material fabricated via selective laser melting. Key Eng. Mater..

[10] Yang P., Deibler L.A., Bradley D.R., Stefan D.K., Carroll J.D. (2018). Microstructure evolution and thermal properties of an additively manufactured, solution treatable AlSi10Mg part. J. Mater. Res..

[11] Strumza E., Yeheskel O., Hayun S. (2019). The effect of texture on the anisotropy of thermophysical properties of additively manufactured ALSi10Mg. Addit. Manuf..

[12] Maity T., Chawake N., Kim J.T., Eckert J., Prashanth K.G. (2018). Anisotropy in local microstructure—Does it affect the tensile properties of the SLM samples?. Manuf. Lett..

[13] Liu X., Zhao C., Zhou X., Shen Z., Liu W. (2019). Microstructure of selective laser melted AlSi10Mg alloy. Mater. Des..

[14] Prashanth K.G., Scudino S., Klauss H.J., Surreddi K.B., Lober L., Wang Z., Chaubey A.K., Kuhn U., Eckert J. (2014). Microstructure and mechanical properties of Al-12Si produced by selective laser melting: Effect of heat treatment. Mat. Sci. Eng. A.

[15] Liu B., Li B., Li Z. (2019). Selective laser remelting of an additive layer manufacturing process on AlSi10Mg. Results Phys..

[16] Hyer H., Zhou L., Park S., Gottsfritz G., Benson G., Tolentino B., McWilliams B., Cho K., Sohn Y. (2020). Understanding the laser powder bed fusion of AlSi10Mg alloy. Metallogr. Microstruct. Anal..

[17] (2014). Material Data Sheet: EOS Aluminium AlSi10Mg.

[18] Bergman T.L., Lavine A.S., Incropera F.P., DeWitt D.P. (2011). Fundamentals of Heat and Mass Transfer.

[19] Thijs L., Kempen K., Kruth J., Humbeeck J.V. (2013). Fine-structured aluminium products with controllable texture by selective laser melting of pre-alloyed AlSi10Mg powder. Acta. Mater..

[20] Wang L., Wang S., Hong X. (2018). Pulsed SLM-manufactured AlSi10Mg alloy: Mechanical properties and microstructural effects of designed laser energy densities. J. Manuf. Process..

[21] Kempf A., Hilgenberg K. (2020). Influence of sub-cell structure on the mechanical properties of AlSi10Mg manufactured by laser powder bed fusion. Mat. Sci. Eng. A.

[22] Maamoun A.H., Xue Y.F., Elbestawi M.A., Veldhuis S.C. (2019). The effect of selective laser melting process parameters on the microstructure and mechanical properties of Al6061 and AlSi10Mg alloys. Materials.

[23] Kurz W., Bezençon C., Gäumann M. (2001). Columnar to equiaxed transition in solidification processing. Sci. Technol. Adv. Mater..

[24] Liu S., Zhu H., Peng G., Yin J., Zeng X. (2018). Microstructure prediction of selective laser melting AlSi10Mg using finite element analysis. Mater. Des..

[25] Efunda Website. http://www.efunda.com.

